# Cologne Consensus Conference: providers in accredited CME/CPD 11–12 September 2015, Cologne, Germany

**DOI:** 10.3402/jecme.v5.31437

**Published:** 2016-04-05

**Authors:** Julie Simper

**Affiliations:** ^a^ International CME/CPD Consulting, Amsterdam, The Netherlands

**Keywords:** CME, CPD, provider, COI, bias, Europe, accreditation, educational event

## Abstract

On 11–12 September 2015, the fourth annual Cologne Consensus Conference (CCC) was held in Cologne, Germany. The 2-day educational event was organised by the European Cardiology Section Foundation (ECSF) and the European Board for Accreditation in Cardiology (EBAC), a specialty continuing medical education–continuing professional development (CME—CPD) accreditation board of the European Union of Medical Specialists (UEMS). The conference was again planned in cooperation with an impressive group of international organisations and faculty members representing leading European and North American institutions. Each year, the CCC is organised around a specific topic area. For the conference's fourth iteration, the providers in accredited CME/CPD were the focus. The CCC 2015 set out to share ideas, discuss concepts, and increase collaborations amongst the various groups. This report provides a summary of the presentations and discussions from the educational event.

## Introduction

On 11–12 September 2015, the fourth annual Cologne Consensus Conference (CCC) was held in Cologne, Germany. The 2-day educational event was organised by the European Cardiology Section Foundation (ECSF) and the European Board for Accreditation in Cardiology (EBAC), a specialty continuing medical education–continuing professional development (CME–CPD) accreditation board of the European Union of Medical Specialists (UEMS). The conference was again planned in cooperation with an impressive group of international organisations and faculty members. However, this year the breadth of representation extended beyond the traditional European and North American institutions to include Qatar's Council for Healthcare Practitioners. Each year, the CCC focuses on a specific topic area. For the conference's fourth iteration, providers in accredited CME/CPD were the focus. This report provides a summary of both the presentations and discussions from this thought-provoking educational event.

### Day 1

Background

- Introduction
- Is CME/CPD Commercial Business: What Does EU Law Say?
  What Do Providers Offer: Present and Future?
- European Society of Cardiology (ESC)
- European Respiratory Society (ERS)
- Guidelines International Network (G-I-N)
- Cochrane Collaboration
- Drug Commission of the German Medical Association (AkdA)
- Good CME Practice Group

### Day 2

What Do Providers Offer: Present and Future? *(continued)*

- Sharing Experiences: Provider Accreditation in North America (US and Canada)
- Advancing ACCME Accreditation in Support of CME/CPD
  What Do Differences in Healthcare Systems Contribute?
- Qatar
- Germany
- Future Directions for CPD Accreditation: the Who, What, and How

## Background

*Chairs: Prof. Heinz Weber, MD, PhD, Chairman, ECSF Foundation Council, Cologne, Germany*

*Craig Campbell, MD, Director CPD, Royal College of Physicians and Surgeons of Canada, Ottawa, Canada*

### Introduction

*Speaker: Prof. Reinhard Griebenow, MD, PhD, Conference Chair; Chairman, EBAC Advisory Committee, Cologne, Germany*

Prof. Reinhard Griebenow, Conference Chair, set the stage for the 2-day educational meeting by proposing five theses that would serve as guiding principles encountered throughout the rest of the conference.

### Thesis 1: CPD should be provided by an expert physician to expert physicians

Prof. Griebenow began with the foundational concept that “it is from immersion in practice that effective CPD arises.” Thus, it is the expert physicians themselves who are the most qualified to educate fellow physicians. However, Prof. Griebenow quickly tempered this statement by recognising that the highest levels of quality are not achieved unaided; support is needed whether that be logistical, administrative, or even with content, when beyond the expert's scope. He likened this to world-renowned violinist Johannes Strauss creating his art supported by a symphony orchestra with its variety of instruments. Without this dynamic collaboration, would the soloist achieve the same levels? This orchestra is to the soloist as the provider is to the expert physician. The resulting questions then become: Which support roles and groups are needed, and which core elements can only the expert/soloist bring?

### Thesis 2: “Existence determines consciousness” (Karl Marx)

Beyond recognising the need for provider support to the expert physician, Prof. Griebenow went on to stress the importance of examining the conditions and context of both groups; not only of the provider but also of the physicians themselves. Are there conflicts of interest or other biases that might be willingly or unwillingly included in the CME/CPD activities?

### Thesis 3: With regard to the organisational framework (including finance), it is no longer the “who” but the “what” and the “how”

With this postulate, Prof. Griebenow stepped away from focusing on which groups, whatever their nomenclature, are involved in CME/CPD to underline the need to assess their actions: What are they doing and how are they doing it? This would be a core concept throughout the conference when trying to define a provider and judge the quality thereof.

### Thesis 4: Participants seem to be more focused on the single CPD activity than on the overall provider performance (including quality, independence, and organisation)

CPD should be longitudinal, fuelling the physician's practice over time to achieve real learning and change. Providers should therefore be assessed in the same way: Are they implementing certain standards and practices over time to ensure quality and consistency in the education delivered?

### Thesis 5: For further delineation of the “what” and “how,” we need to define standard operating procedures (SOPs) as well as standard operating components (SOCs) for the planning and delivery of CPD

With his final thesis, Prof. Griebenow went to the heart of the discussion and the purpose of the conference. Studies and practical experience show that there is a transfer of many CME/CPD organisational elements from physician control to non-physician groups. Supportive of this reality, Prof. Griebenow went on to introduce just some of the questions generated by this increasing delegation of tasks.

Prof. Griebenow closed his introductory session by underlining that these concepts and more would be explored throughout the conference.

### Is CME/CPD Commercial Business: What Does EU Law Say?

*Speaker: Helmut Koenig, Beiten Burkhardt Lawyers, Dusseldorf, Germany*

The CCC generally approaches the topic at hand by also including points of views from those working outside the traditional CME/CPD community. The year 2015 was no exception with Mr. Koenig contributing his over 25 years of experience as a tax advisor and public auditor to provide a legal perspective on the fundamental question: Is CME/CPD considered a commercial business? He began by outlining two key framing points.

### Determination of Position: Services of General Interest

This includes a broad range of different types of activities, from the big network industries (energy, postal services, transport, and telecommunications) to health, education, and social services. The European Parliament believes that certain services of general interest should be excluded from the scope of competition rules. However, it also considers that it is neither possible nor relevant for common definitions of services of general interest to be drawn up.

### Point of Reference: Direct and Value-Added Taxes (VAT)

Mr. Koenig briefly described several articles from the Organisation for Economic Co-operation and Development (OECD) Model Tax Convention with respect to taxes on income and on capital with regard to direct taxes and VAT, ultimately exposing the lack of common law with regard to direct taxes on income and that these are subject to national legislation. He also exposed how “The law on value-added tax is not always readily understandable” (Opinion of Advocate General of European Court of Justice Prof. Dr. Juliane Kokott, 2013) and that VAT is based on Council Directive 2006/112/EC which is mandatory for all EU member states. However, this directive offers room for interaction with national legislation as far as tax exemptions are considered.

### Judgements of the European Court of Justice

The European Court of Justice is the competent court for judgements on EU-VAT topics and Mr. Koenig briefly described cases challenging some of the legal and fiscal regulations. He explained how some judgements even altered the national VAT legislation of EU member states.

In summary, based on the legal sources examined in preparation for his presentation, Mr. Koenig concluded that CME/CPD does have to be considered a commercial business. He also warned that VAT is the most dangerous tax for enterprises, institutions, associations, and public bodies providing CME/CPD. The tax authorities tend to treat even the smallest of mistakes or omissions as tax fraud; which can result in criminal prosecution of the individuals involved. However, Mr. Koenig ended his presentation with a positive note that the EU-VAT law does offer some legal room for “design” of tax situations. But he provided the practical advice that any such fiscal designing will take time and planning and should be done under the counsel of legal and fiscal expert advisors.

## What Do Providers Offer? Present and Future

*Chairs: Graham McMahon, MD, MMSc, President and CEO, Accreditation Council for CME, Chicago, USA*

*Peter Mills, MD, BM, BCh (oxon), BSc, MA, FRCP, Member, ECSF Board, Cologne, Germany, London, United Kingdom*

### European Society of Cardiology (ESC)

*Speaker: Peter Kearney, MD, Chair of the Cardiovascular Division, Cork University Hospital, Cork, Ireland*

Calling in via Skype, Dr. Kearney provided an introduction to the European Society of Cardiology (ESC) CME/CPD activities. He began by explaining that ESC engages in both education (lifelong learning) and training in cardiovascular medicine with the aim to demonstrate a positive impact on outcomes. He outlined the vast array of CME/CPD offerings ranging from global live events to a multitude of web-based education and resources. Dr. Kearney went on to emphasise that the ESC is evolving its approach to medical education to ensure the offerings are credible, needs driven, evidence based, and relevant to members. He described three key initiatives in support of these goals.

### ESCel=Online Curriculum ESC Education and Training Platform

ESC has developed its online platform ESCel to deliver curriculum-based education and training in general cardiology, and subspecialties including acute cardiac care, cardiovascular imaging, heart failure, heart rhythm, percutaneous intervention, and prevention & rehabilitation.

### ESC Education Conference=Continuous Engagement

Each year, the national directors of training and education of the ESC member countries meet, network, and discuss challenges and themes around medical education and training.

### Needs Assessment Methodology=Credibility and Identification of Gaps

ESC has also mandated a third party research organisation to run studies and formal needs assessments using a robust methodology and with ethics approval from an international review board.

Dr. Kearney also touched on the current accreditation challenges wherein the EBAC only provides credits for live events or e-learning courses that are at least 1 hour long. The result is that the ESC does not submit many of its novel educational activities to EBAC for credit; for example, short activities or complete programmes on ESCel.

**Figure 1:**
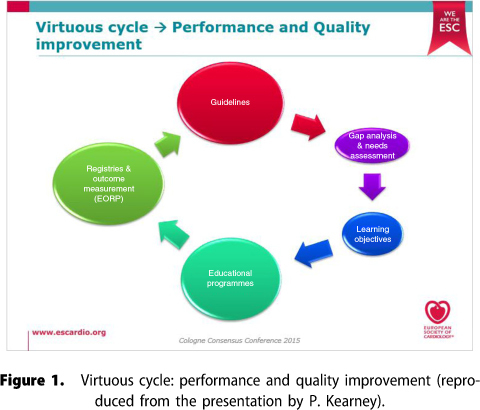
Virtuous cycle: performance and quality improvement (reproduced from the presentation by P. Kearney).

In summary, Dr. Kearney said that the ESC is in a period of evaluation and transition of its CME/CPD activities (Figure 1). It is seeking to evolve from a strongly science-centred approach to focus increasingly on learning and education. This evolution is expert led, curriculum based, guidelines based, case based, patient centred, and especially needs driven and evidence based.

### European Respiratory Society (ERS)

*Speaker: Prof. Daiana Stolz, MD, MPH, Clinic for Pulmonary Medicine and Respiratory Cell Research, Basel, Switzerland*

Representing the European Board for Accreditation in Pneumology (EBAP), Prof. Stolz began by outlining the EBAP mission and constitution while explaining that the board accredits live events, printed materials, and e-learning programmes for customers ranging from scientific societies to medical education companies (MedEc). She went on to introduce key ERS educational planning elements such as topic selection methodologies, speaker selection, conflict of interest management, transparency efforts, and outcomes measurement activities.

Prof. Stolz directly addressed the heart of the conference topic by exploring some of the differences between scientific societies and professional congress organisers (PCO) or MedEc. According to Prof. Stolz, the key differentiators are as follows.

### Subject-Specific Expertise

Scientific societies bring together physician experts in a specific and targeted area, serving as the source of expertise for CME/CPD activities. In contrast, PCOs or MedEcs typically work in a variety of areas, not exclusively medical.

### Lifelong Learning

Furthermore, scientific societies approach CME/CPD from a lifelong learning perspective, aiming to support their members longitudinally throughout their careers, whereas PCOs and MedEcs take a cross-sectional approach to the activities they provide.

### Non-Profitability

Prof. Stolz posited that the non-profit status of a scientific society is a safeguard against bias, while the profit-orientated business model of PCOs and MedEcs presents greater risk of fundamental conflicts of interest. She went on to outline several EBAP efforts safeguarding against such risk, including reviewing contracts with sponsors when possible, checking the amounts received by faculty, focusing on the extent of influence of the sponsor on content, and sending onsite rapporteurs to live events.

### Guidelines International Network (G-I-N) & American College of Physicians (ACP)

*Speaker: Amir Qaseem, MD, PhD, MHA, FACP, Vice President, Clinical Policy, American College of Physicians, Philadelphia, USA; Immediate Past Chair, Guidelines International Network, Berlin, Germany*

Dr. Qaseem began by introducing the Guidelines International Network (G-I-N) as an international organisation founded in 2002 and comprising 100 organisations and 131 individual members from 48 countries. The mission of G-I-N is to lead, strengthen, and support collaboration and work within the guideline development, adaptation, and implementation community. The network supports evidence-based healthcare and improved health outcomes by reducing inappropriate variation throughout the world.

Before examining the role of guidelines in CME/CPD, Dr. Qaseem underlined some of the issues surrounding disclosure of financial and intellectual interests and management of conflicts of the guideline developers and included some recent examples of where bias (real or perceived) lessened the validity of the guidelines. He stressed the importance of independence in guideline creation given that guidelines not only influence clinical actions and decisions in patient care but are also used for creating performance standards and reimbursement policy. He discussed that conflicts can only be managed, but not resolved, especially those in the past. The discussions closed with the recommendation that given the important role that highly rated guidelines play in medicine, providers should account for conflicts to promote trust, preserve scientific integrity, and reduce biases.

### Cochrane Collaboration

*Speaker: Prof. Gerd Antes, DSc, Director, German Cochrane Centre, Centre for Medical Biometry and Medical Informatics, Freiburg, Germany*

**Figure 2:**
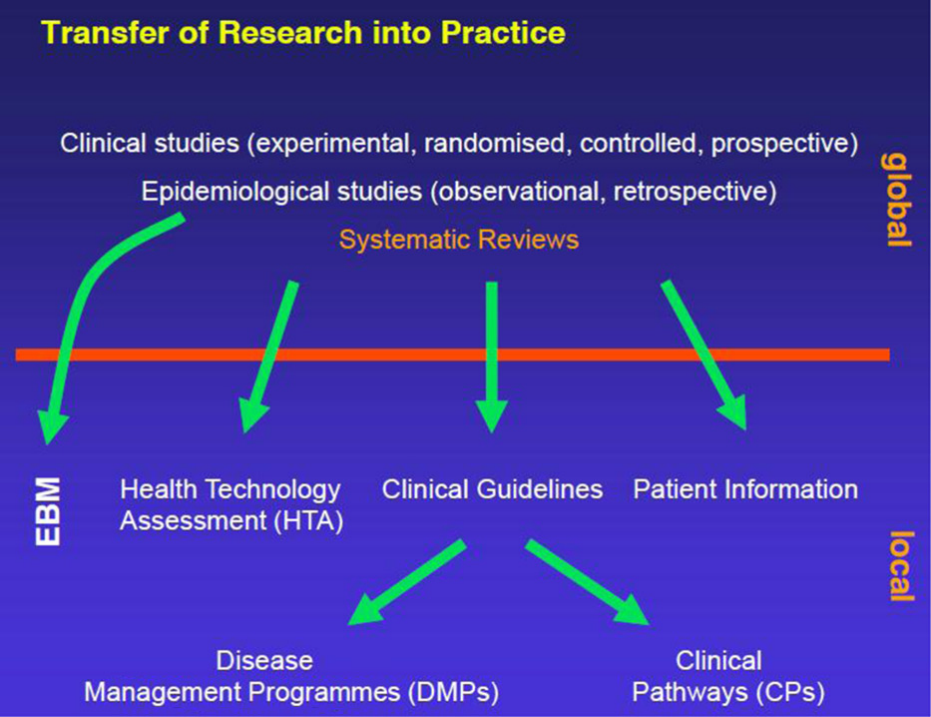
Transfer of research into practice (reproduced from the presentation by G. Antes).

Prof. Antes began by introducing the Cochrane Collaboration as a global independent network of researchers, professionals, patients, caregivers, and people interested in health, with the mission to promote evidence-informed health decision-making by producing high-quality, relevant, accessible systematic reviews and other synthesised research evidence. Prof. Antes went on to offer a definition of evidence-based medicine and affirmed the key role therein of values, clinical expertise, and external evidence, the latter being the focus of his presentation. He went on to provide an overview of the transfer of research into practice (see Figure 2). Amid this system of knowledge accumulation and dissemination, Prof. Antes discussed the importance of independent systematic reviews that:

- Frame the question
- Perform a systematic search for evidence from relevant trials and studies
- Summarise and offer a quantitative synthesis (if possible)
- Interpret and put in context
- Update regularly

Ultimately, systematic reviews should include all relevant data from all participants in all relevant trials. However, according to Prof. Antes, this is impossible because of selective reporting of trial data: omission of entire trials, incomplete or biased information within published trials, and discrepancies between quantitative results and interpretation. He also touched upon the limiting role of English being the primary language in knowledge creation and dissemination, as only 5% of the world population is Anglophone. The non-existing stopping rule addresses the generation of evidence, that is, when is the existing knowledge (evidence) sufficient for decision-making and therefore no further trials are needed? In which case, all further trials would be unethical because unnecessary trials are unethical. This implies the necessary condition that (1) all existing trials are known with full information and (2) every planning of a new trial is based on complete knowledge of the existing evidence.

In summary, he acknowledged the limited impact of current requirements around trial registration, publication, and full access to trial reports and trial data. However, he did add that major organisations, including the Cochrane Collaboration, are working to address these limitations. Prof. Antes ended by relating this to CME/CPD:

- Summarising and disseminating evidence/knowledge is a key ingredient for better translation into practice.
- Systematic reviews are *the* technology for accumulating evidence.
- The Cochrane Collaboration performs approximately 25% of all systematic reviews and offers a rich spectrum of content, methodology, and technology for CME/CPD (Note: EBAC is working to provide free access to the Cochrane database for its CME/CPD providers).
- There is an accelerating growth of the number of systematic reviews, often resulting in more than one systematic review on the same question. Although claiming to follow similarly rigid methodologies, the different reviews may come out with different results. This challenges the user to judge which one is correct. Therefore, a basic understanding of the principles and the methodology of systematic reviews is essential for judging and selecting reliable knowledge. Prof. Antes recommends this should be a compulsory building block of CME/CPD.

### Drug Commission of the German Medical Association

*Speaker: Prof. Klaus Lieb, MD, Drug Commission of the German Medical Association, Berlin, Germany; University Medical Centre, Mainz, Germany*

Prof. Lieb presented on behalf of the Drug Commission of the German Medical Association (DCGMA), which is a scientific expert committee for drug-related matters consisting of 40 full members and approximately 150 associate members from all areas of medicine and pharmacy.

**Figure 3:**
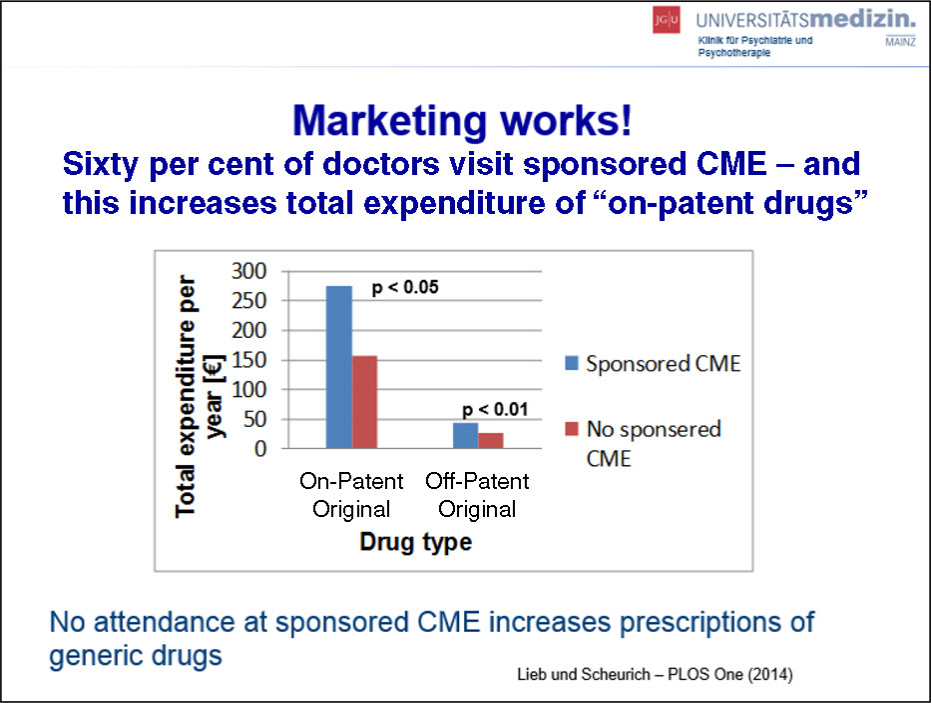
Sponsored CME and prescriptions (reproduced from the presentation by K. Lieb).

Prof. Lieb began by framing the conversation with a definition and examples of Conflict of Interest (COI) and recognising that academia–industry collaboration has indeed resulted in countless innovations. However, he also reminded the audience that marketing is the primary interest of industry and industry data are frequently, if not always, biased in favour of the promoted drug. He went on to provide data on industry marketing spend, including amounts spent on CME, noting that globally, approximately 33% of all accredited CME is funded by drug companies. He also provided data positively correlating physician attendance at sponsored CME and resulting prescriptions of “on-patent drugs” versus prescriptions of the generic alternatives (see Figure 3). He went on to outline further influences, direct and indirect, of industry on CME, including agenda setting, publication bias, influence on the study protocol, and ghost writing of medical articles.

In view of the concern about these data, Prof. Lieb discussed efforts of the DCGMA to provide unbiased CME. He outlined key challenges for so doing, including how to avoid COI, find experts with no or little industry contacts, manage the influence of non-financial COI, evaluate objectivity and independence, and finance unbiased CME when doctors are used to participating in CME for free. Prof. Lieb outlined current DCGMA criteria for CME which they consider to be “unbiased by influences of pharmaceutical industry.”

- Independent, non-industry financing
- Selection of “independent” speakers
- Commitment of presenters to the objective presentation of training contents
    ° Alternative therapeutic options have to be presented and comparatively assessed
    ° Representation of data must take into account systematic reviews, meta-analyses, and reviews by independent institutions
    ° The advantages and disadvantages of new treatment options as well as the limitations of study results have to be discussed
- Commitment of the scientific chair to conduct an evaluation of the event, including questions on bias
- Declaration and discussion of COI by lecturers, scientific chairs, and presenters

Prof. Lieb closed by recognising the challenge to implement such strict criteria across the broader CME global community. However, he hoped that these initial efforts by the German Medical Association would serve as a stimulus to positive change on a broader scale.

### Good CME Practice Group

*Speaker: Eugene Pozniak, Siyemi Learning, Manchester, United Kingdom*

Mr. Pozniak began his presentation by asking the question: Is CME/CPD a commercial business? Instead of providing an answer, he challenged conference attendees by asking further questions: Is treating patients a commercial business? Is Médecins Sans Frontières a commercial business? Is being a parent a commercial business? After all, suggested Mr. Pozniak, all of these activities require money to be exchanged in order to function. Therefore, he proposed to reframe the initial question as: Is CME/CPD a *professional* business?

To answer this question, Mr. Pozniak offered a snapshot of CME providers in Europe along with a list of the various roles and responsibilities (regulatory and CME compliance, legal, project management, educational design, outcomes evaluation, etc.) that any provider must fulfil, regardless of the type of provider (academic, hospital, medical society, independent company, etc.). He referred back to Prof. Griebenow's introductory presentation and reaffirmed the role of the provider as a critically important support to the expert physician, encouraging the non-physician professional provider to be the preferred party to be responsible for many CME/CPD organisational elements. Key to the validity of this concept is appreciation of the many rules that providers must follow when developing educational activities from regulatory organisations such as the accrediting bodies, national/European legal entities and laws (anti-bribery, foreign corrupt practices, tax regulation, etc.), industry associations (European Federation of Pharmaceutical Industries and Associations, Eucomed, national, etc.), or individual companies.

Further, Mr. Pozniak stressed that many CME/CPD providers are also self-motivated to be high-quality and ethical professional partners to the expert physician. To illustrate this point, Mr. Pozniak introduced the Good CME Practice Group as a collection of 14 providers with a positive track record in and strong commitment to European CME. Providers must apply to join, and reaffirm annually, by demonstrating their independence from industry, compliance with leading accreditation bodies, professionalism in faculty management, and quality delivery to the learner audience. He briefly outlined some of the key initiatives of the Good CME Practice Group: promotion of the four core principles^1^ of appropriate education, balance, transparency, and effectiveness; advocacy on behalf of providers; and provider collegiality and sharing of best practices. He continued by underlining the importance of CME/CPD accreditation to providers as one way of demonstrating this independence, professionalism, and quality. Mr. Pozniak also provided his thoughts on those organisations controlling standards and providers, and highlighted recent government interventions resulting from things having gone wrong. Mr. Pozniak concluded with thoughts on the future role of the provider:

- Providers need the desire, rules, and guidance to do their jobs well.
- Providers need to demonstrate to others the good work that is being done.
- Providers need to be formally recognised as an integral part of the CME programme.
- Providers need to be accountable not only for their own actions but also for those of the planning committee, providing leadership when appropriate.
- Providers need to recognise that a medical practitioner must always be responsible for the content.

### Sharing Experiences: Provider Accreditation in North America (US and Canada)

*Speakers: Jennifer Gordon, MEd, CAE, Associate Director, CPD, Royal College of Physicians and Surgeons of Canada, Ottawa, Canada; Kate Regnier, MA, MBA, Executive Vice President, Accreditation Council for CME, Chicago, USA*

Ms. Gordon and Ms. Regnier began by outlining the goals of their presentations which were to share experiences of two provider-based accreditation systems. They did this by each providing a comprehensive overview of their respective systems across key areas as summarised in the following tabular column. [Table T0001]

**Table T0001:** 

Royal College of Physicians and Surgeons of Canada	Accreditation Council for Continuing Medical Education

History/background	
Established early 2000, voluntary, hybrid provider/activity Principles: based on a set of standards; demonstrates accountability/fairness, mark of educational quality; promotes continuous quality improvement Governance: Royal College CPD Accreditation Committee sets the standards for accredited providers in national specialty societies and individual activities, overseen by the Royal College CPD Committee Committee on Accreditation of CME (CACME) sets the standards for accredited university offices of CME Positioned within the larger Maintenance of Certification Program framework for CPD activities	Created in 1981, comprising seven member organisations representing key US medical organisations Voluntary self-regulation, “for the profession, by the profession” Principles: linked to quality/safety, effective in improving practice, independent of commercial interests, based on valid content Bylaws/primary functions: accredit institutions offering CME, develop criteria, develop methods for measuring the effectiveness of CME
Provider eligibility	
Canadian national organisation Aligned with a Royal College specialty, sub-speciality, or area of focused competency Physician organisation	Offer CME for physicians on a regular/recurring basis Valid content Not be a commercial interest (according to formal ACCME definition) Not provide CME that advocates unscientific modalities Cannot promote treatment known to be ineffective or whose risks outweigh benefits
Initial/reaccreditation process	
Accreditation cycle 3–6 years, same process for initial application as for reapplication Coaching/submission of application Independent peer review 3-hour teleconference Draft of reviewers’ report CPD Accreditation Committee Final report to applicant Post-review teleconference Action plans/interim reports	Whether initial or reaccreditation, decision is based on three sources of data: Self-study report Evidence in performance-in-practice Accreditation interview Timelines for data collection, decision-making, and feedback/decision notification: Initial accreditation: after determining eligibility, 12 months Reaccreditation every 2–4–6 years depending on accreditation status
Standards/criteria	
Twelve provider standards organised into three sections: purpose/mission, educational planning/implementation/evaluation, sustainability Each standard includes the standard/anchor/description, evaluation criteria, documentation requirements, self-study questions Document checklist and glossary of terms are included	Combination of provider/programme-level and activity-level standards designed conceptually around the Plan–Do–Study–Act quality improvement methodology and engagement with the environment Criteria 1–13 required for accreditation, 16–22 for accreditation with commendation Additional policies for verifying physician participation, maintaining records, and submitting reports/fees
Education/support	
Strong organisational commitment to and investment in provider education and support via educational conferences, newsletters, website resources, tools, best practices, and staff support for questions/requests for information.	
Monitoring: fees, reports, complaints, data	
Action plans (typically first year of cycle) to address standards deemed non-adherent or partially adherent Interim reports (by mid-cycle) demonstrating how the providers implemented their action plans Royal College CPD unit provides feedback and reassesses standards as required (focus on continuous quality improvement) No direct fees; system funded by the Royal College	Progress reports to address areas of non-compliance identified in reaccreditation (focus on continuous quality improvement) Online complaints process Focused surveys Annual reporting online in Program and Activity Reporting System (PARS) Fee schedule variable, for example, initial accreditation $8,000, annual accreditation $5,400
International agreements	
Credit conversion to/from: AMA PRA Category 1 Credit Agreement European Union of Medical Specialists (UEMS)	Recognition of Royal College's CPD accreditation system as substantially equivalent Recognition of Committee on Accreditation of CME of Canada as substantially equivalent Recognition of the European Board for Accreditation of Cardiology as substantially equivalent Recognition of the Oman Medical Specialty Board of the Ministry of Health of the Sultanate of Oman as substantially equivalent
Future	
Accreditation alignment across the medical education continuum Accreditation management system (IT) National CPD provider standards National standard for support of accredited CPD activities Evolved eligibility requirements	Commendation criteria Alignment with other systems Needs of stakeholders (e.g. maintenance of certification, licensure, quality, safety, the continuum of medical education) Attention to education research Relationships with learners Interprofessional continuing education Global relationships (accreditors and providers)

### Advancing ACCME Accreditation in Support of CME/CPD

*Speaker: Graham McMahon, MD, MMSc, President and CEO, Accreditation Council for CME, Chicago, USA*

Dr. McMahon, the new President and CEO of ACCME, participated for the first time in the CCC by recognising the major challenges faced by CME due to realities like the lack of engaged clinician leaders and the shifting expectations of learners and confusing and diverse credit systems nationally and internationally. He stressed the need for better alignment of effective pedagogy and accreditation requirements with how physicians learn and practise. Dr. McMahon quickly moved on to present the key opportunities he believes will be created when the health system invests in CME providers.

### Meeting the Learners’ Needs

With increased investment, CPD providers will be better able to meet the needs of learners who, beyond simply obtaining credits, want a learning system that is:

- Relevant based on local/community data, the learner's data and/or the learner's expressed need
- Efficient and respectful of their time, easy to access, and flexible
- Effective and moving from passive learning to learning based on adult learning principles (participatory, skills-based, opportunities for practice and feedback)
- Rewarding by meeting a need, creating opportunities, providing easily accessible credit transcripts and socialising with colleagues
- Personalised to meet individual needs in the right format, at the right level, and with the right materials

### Delivering Value to the System

**Figure 4:**
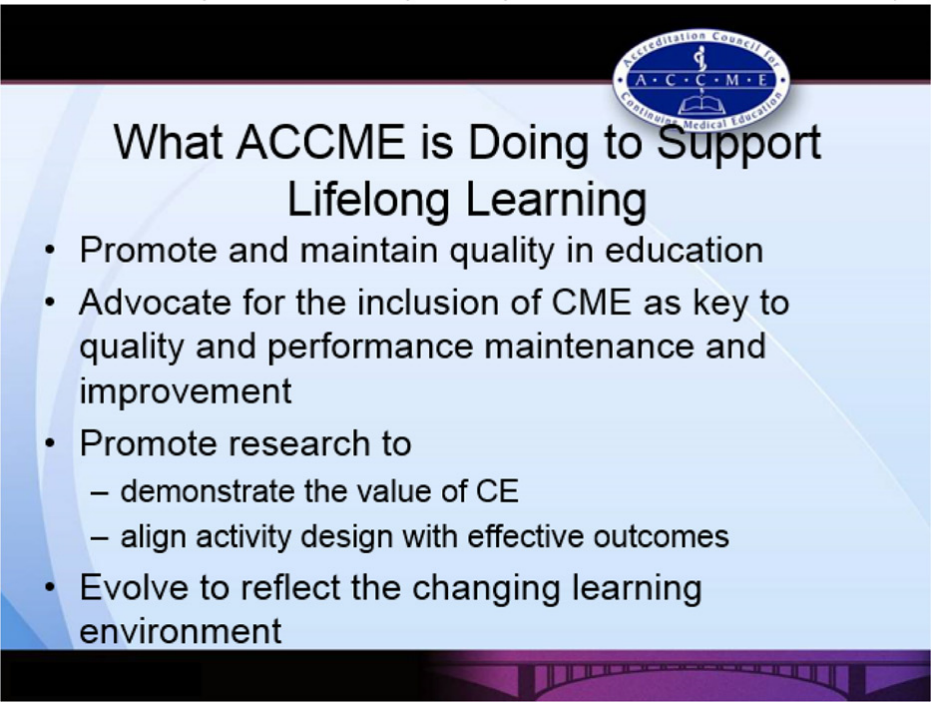
What ACCME is doing to support lifelong learning (reproduced from the presentation by G. McMahon).

When better meeting the needs of learners, Dr. McMahon explained how he sees CME as a key asset that is often effective, quality focused, capacity building, team building, and impactful. It can minimise loneliness, burnout, errors and turnover and help move practitioners from simply “knowing” something to actually “doing” and integrating performance into practice. He also explored how accredited providers might play a role in better integrating and automating points/credits required for credentialing, licensing, and certification. He again stressed, however, the importance of CME providers offering effective pedagogy, using what works for true learning and change, rather than just what is comfortable, convenient, and familiar. Beyond this, he recognised that CME needs to continually develop toward being more consistently based on identified needs, as well as toward being longitudinal, individualised, interprofessional, and independent in nature. He also discussed the importance of CME supported by research. He closed by outlining what the ACCME is doing to support lifelong learning (see Figure 4), including a brief introduction of the revised accreditation with commendation criteria currently under consideration.

## What Do Differences in Healthcare Systems Contribute?

*Chairs: Prof. Reinhard Griebenow, MD, PhD, Conference Chair; Chairman, EBAC Advisory Committee, Cologne, Germany*

*Kate Regnier, MA, MBA, Deputy Chief Executive and Chief Operating Officer, ACCME, Chicago, USA*

### Qatar

*Speaker: Prof. Samar Aboulsoud, MBBCh, MSc int. med, MD, MSc Med Ed, FHEA, MAcadMEd, Manager, Accreditation and Health-Profession Education Department, Qatar Council for Healthcare Practitioners, Qatar*

Dr. Samar Aboulsoud joined the conference via Skype to share recent experiences of developing Qatar's national CPD framework and accreditation system. Although the system is still in its infancy, Qatar is already a member of the International Academy of CPD Accreditors.

### Background

Dr. Aboulsoud found it important to note the uniqueness of Qatar's healthcare workforce which is primarily expatriate from a wide range of countries with variable educational backgrounds and work experience. In addition to these disparities, prior to 2013, licensing was managed by a department of the Supreme Council of Health that oversaw the licensing of healthcare practitioners primarily in the private sector. Further, up until 2016, participation in CPD was optional and done on an institutional level.

### Introduction to the Qatar Council

This would all change with the introduction in 2013 of a regulatory and accreditation body, the Qatar Council for Healthcare Practitioners (QCHP). The organisation was created as part of the national health strategy to regulate the healthcare practice of all practitioners in all sectors, as well as oversee accreditation and health profession education from undergraduate to graduate and on to CME/CPD. The intention was to set CME/CPD standards and a means of enforcing them through the accreditation process, while linking CPD to licensure.

### Creating a New CME/CPD System

The QCHP recognised from the outset the value of international collaborations and submitted a request for proposal from established accrediting bodies to assist with the development process. The Royal College of Canada International was selected as the organisation with whom to partner. With a detailed project roadmap and a closely collaborative, reiterative development process, a CPD framework for healthcare practitioners and a CPD accreditation system were created.

### The Practitioner's Perspective: CPD Programme for Healthcare Practitioners

**Figure 5:**
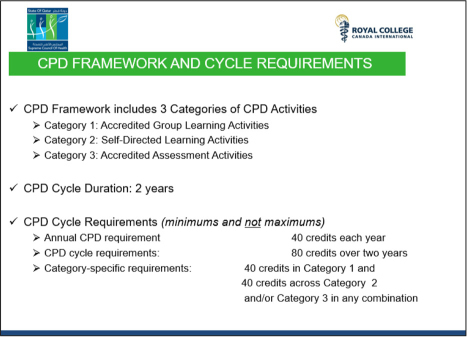
CPD framework and cycle requirements (reproduced from the presentation by S. Aboulsoud).

Under the new system, participation is mandatory for all healthcare practitioners, based on a single set of standards, linked to licensure, and this on a national level. See Figure 5 for the CPD framework and cycle requirements. This system is expected to be launched in March 2016 and will allow practitioners a phased-in approach.

### The Provider's Perspective: National CPD Accreditation System

Dr. Aboulsoud went on to explain that Qatar's CPD accreditation system would be a national, unique system unified for all healthcare practitioners; while being a hybrid system embracing both provider-based and activity-based accreditation. She went on to explain that much like the Canadian system from which it was modelled, the provider-based system would promote continuous quality improvement of its providers, united in a learning community. She went on to outline the provider standards, similar to those of the Canadian system (see presentation summary of J. Gordon above). She closed this portion of the presentation by explaining that the activity-based CPD accreditation elements of the Qatar system would also comprise standards for all individual activities and that these activities would be reviewed and approved by the QCHP Accreditation Department (QCHP-AD).

In closing, Dr. Aboulsoud underlined the importance of stakeholder engagement and scholarly collaboration throughout the process; whether working with the Royal College of Canada International or the extensive CPD taskforce comprising representatives from government, the private sector, and healthcare practitioners of all types.

### Germany

*Speaker: Prof. Reinhard Griebenow, MD, PhD, Conference Chair; Chairman, EBAC Advisory Committee, Cologne, Germany*

*Slide Author: Dirk Schulenburg, JD, Chamber of Physicians Northrhine, Dusseldorf, Germany*

**Figure 6:**
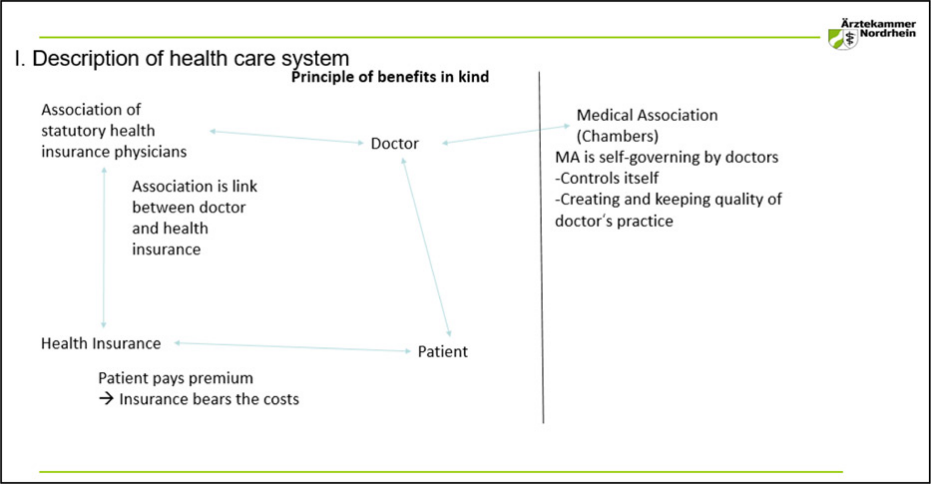
Description of German healthcare system (reproduced from the presentation by D. Schulenburg, R. Griebenow).

Presenting Mr. Schulenburg's slides, Prof. Griebenow began by providing some background on the healthcare system. In Germany, citizens are obligated to have some form of legal health insurance; with social insurance funding 90% of the population's healthcare and the other 10% obtaining private insurance. With such coverage, patients do not pay the costs directly. Payment is made by insurance companies through the agency of an association of statutory health insurance physicians, of which doctors are members and through which they bill for services. Prof. Griebenow went on to provide some insight into the patients’ right to choose their own practitioner, receive accurate qualified therapy, and have costs covered by the legal health insurance, to such an extent that even those therapies lacking substantive data on their efficacy are covered (“Santa Claus” decision of the Federal Supreme Court). Prof. Griebenow then described the position of the doctor within this system, and more specifically the physician's obligation of membership within their regional Medical Association or Chamber. Medical Associations are self-governing extensions of the government's Ministry of Health overseeing a broad scope of physician activities, including licensure, maintenance of licensure and CPD, with the Chamber being both a provider and an accrediting body (see Figure 6).

Until 2004, CME/CPD was only encouraged under a professional code of conduct. However, this was deemed insufficient and left no room for sanctions; so in 2004, documented participation in CME/CPD became mandatory for established physicians, specialists in hospitals, and part-time physicians. The German CPD framework is built on legal regulations, applicable to both the medical associations, as well as the medical professionals themselves. A key criterion of CPD in Germany is that the education must be free of economic interests, with companies disallowed from directly affecting the CPD. However, as Prof. Griebenow explained, there remains an important risk for indirect influence as companies still fund the high cost of CPD, support consultants with positive opinions, and support events with positive content and topics. Because of this risk, and the legal requirement to provide independent CPD, the Chambers, acting as the accrediting bodies, embrace activity accreditation with high levels of control, strong oversight, and a limited degree of variability.

### Future Directions for CPD Accreditation: The Who, What, and How?

*Speaker: Craig Campbell, MD, Director CPD, Royal College of Physicians and Surgeons of Canada, Ottawa, Canada*

Dr. Campbell presented a comprehensive summary of key points presented throughout the 2-day conference before providing his thoughts on future directions.

### 1. Importance of Language – Provider organisations: Are they providers of continuing medical education or continuing professional development?

Dr. Campbell provided the view of CPD as a much broader concept, in which formal CME is but a portion. He discussed the importance of considering CPD as a collective with shared responsibility between the accrediting bodies, providers, expert educators, and individual groups or health teams. Defining and promoting relationships are key in this encompassing definition of CPD.

### 2. Importance of Vision and Mission – Tension within organisations to view CPD as either “a profit centre” or a “value centre”

With reference to the conference's early presentations, this again underlines the continued issues (ethical, relational, financial, and legal) relevant to the consideration of education as a commercial enterprise.

### 3. Importance of Roles and Responsibilities – Who has the final responsibility for CPD?

Reiterating one of the key concepts of the conference, without disputing that the medical profession is ultimately responsible for the CPD delivered, the question remains regarding what activities can and should be delegated to support organisations and logistical teams.

### 4. Importance of Expertise – CPD as an intervention is a form of implementation science

Dr. Campbell likened CPD to knowledge transfer, patient safety, and continuous quality improvement in that it is a complex intervention based on theoretical foundations and an expanding research base with defined skills and abilities. The development of certificate programs for CPD professionals is an indication of this increasingly recognised expertise.

### 5. Importance of Scope – CPD providers are not just developing live events

Practical experience shows that learners are using multiple activities and resources to address their educational needs. In response, CPD providers are offering a growing scope of educational activities, including web-based or blended activities, guidelines, apps, journals, etc. In parallel, Dr. Campbell stressed the importance of CPD accreditation systems as supporters or enablers of educational quality, innovation, and development, beyond the traditional forms of CME.

### 6. Importance of Standards – Are standards a set of rules or a code of conduct?

As the accreditation systems are based on standards as a “mark of quality,” Dr. Campbell underlined that these must be meaningful, educationally relevant, and ethically coherent. He also reminded the audience that as such, they will evolve and change based on application and the evaluation of their impact on educational and clinical outcomes.

### 7. Importance of independence of CME – Complexity of developing content while managing conflicts, bias, and influence

As always, a central question and concern of CME/CPD regarding how to manage industry involvement in medical education and professional development.

### 8. Importance of Relationships – Shifting from silos to models of collaboration and partnerships

As the practice of medicine becomes increasingly complex so does the provision of good quality CME/CPD. In order to achieve common goals; providers are thus challenged to work increasingly together and establish collaborative partnerships beyond those traditionally encountered.

**Figure 7:**
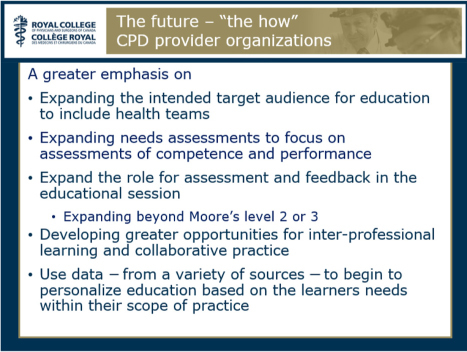
Future directions: “the how” for CPD providers (reproduced from the presentation by C. Campbell).

**Figure 8:**
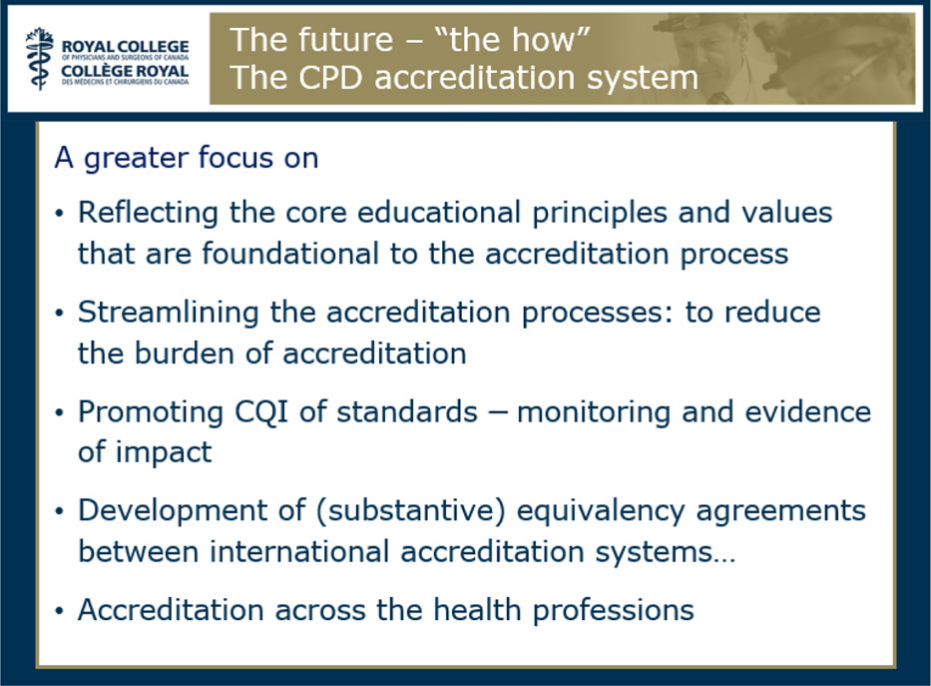
Future directions: “the how”for the CPD accreditation system (reproduced from the presentation by C. Campbell).

After summarising the conference main concepts, Dr. Campbell continued by exploring future directions. He began by saying that in addition to the traditional providers, there is an increasing need for the workplace to support learning and continuing improvement of its physicians and health teams (the “who”). He also urged that CPD providers value and demonstrate strong educational principles and skills with a greater emphasis on interactive learning; moving beyond simply disseminating knowledge and focusing less on the process of education itself and more on the outcomes of education on practice (the “what”). Most importantly, Dr. Campbell also provided several ideas on the “how” to achieve these goals for both the CPD provider (see Figure 7) and the accreditation system (see Figure 8).

## Summary and Conclusion

Over the 2-day conference there was much discussion of the many questions, concerns, and opportunities surrounding CME/CPD provider organisations and their role in the provision of high-quality, effective, and independent medical education. Whether discussing who or what is a provider, the importance of focusing on behaviours versus nomenclature, the challenge of managing COI, or distinguishing the roles and responsibilities of all those involved, the CCC once again provided an exceptional opportunity for international experts and leaders to come together to learn from one another through both formal presentations and lively group discussions.

*The next Cologne Consensus Conference will take place on 16–17 September 2016 in Cologne, Germany, and will focus on Needs Assessments in CME–CPD. For more information on future and past conferences, including presentations and reports, please visit:* http://e-cs-f.org/en/about-ecsf/activities-and-projects.html

## Conflict of interest and funding

The author has not received any funding or benefits from industry or elsewhere to write this report.

## References

[1] Farrow S, Gillgrass D, Pearlstone A, Torr J, Pozniak E, Good CME Practice Group Setting CME standards in Europe: guiding principles for medical education. Curr Med Res Opin. 2012; 28: 1861–1871. 23043468 10.1185/03007995.2012.738191

